# Myofiber organization in the failing systemic right ventricle

**DOI:** 10.1186/s12968-020-00637-9

**Published:** 2020-06-29

**Authors:** Cosimo M. Campanale, Benoit Scherrer, Onur Afacan, Amara Majeed, Simon K. Warfield, Stephen P. Sanders

**Affiliations:** 1 Unit of Perinatal Cardiology, Department of Neonatology, Ospedale Pediatrico Bambino Gesù di Roma, Rome, Italy; 2 Department of Radiology, Boston Children’s Hospital and Harvard Medical School, Boston, MA USA; 3 Department of Cardiology, Boston Children’s Hospital and Department of Pediatrics, Harvard Medical School, Boston, MA USA; 4 Cardiac Registry, Departments of Cardiology, Pathology and Cardiac Surgery, Boston Children’s Hospital, 300 Longwood Ave, Boston, MA 02115, USA. and Department of Pediatrics, Harvard Medical School, Boston, MA USA

## Abstract

**Background:**

The right ventricle (RV) often fails when functioning as the systemic ventricle, but the cause is not understood. We tested the hypothesis that myofiber organization is abnormal in the failing systemic right ventricle.

**Methods:**

We used diffusion-weighted cardiovascular magnetic resonance imaging to examine 3 failing hearts explanted from young patients with a systemic RV and one structurally normal heart with postnatally acquired RV hypertrophy for comparison. Diffusion compartment imaging was computed to separate the free diffusive component representing free water from an anisotropic component characterizing the orientation and diffusion characteristics of myofibers. The orientation of each anisotropic compartment was displayed in glyph format and used for qualitative description of myofibers and for construction of tractograms. The helix angle was calculated across the ventricular walls in 5 locations and displayed graphically. Scalar parameters (fractional anisotropy and mean diffusivity) were compared among specimens.

**Results:**

The hypertrophied systemic RV has an inner layer, comprising about 2/3 of the wall, composed of hypertrophied trabeculae and an epicardial layer of circumferential myofibers. Myofibers within smaller trabeculae are aligned and organized with parallel fibers while larger, composite bundles show marked disarray, largely between component trabeculae. We observed a narrow range of helix angles in the outer, compact part of the wall consistent with aligned, approximately circumferential fibers. However, there was marked variation of helix angle in the inner, trabecular part of the wall consistent with marked variation in fiber orientation. The apical whorl was disrupted or incomplete and we observed myocardial whorls or vortices at other locations. Fractional anisotropy was lower in abnormal hearts while mean diffusivity was more variable, being higher in 2 but lower in 1 heart, compared to the structurally normal heart.

**Conclusions:**

Myofiber organization is abnormal in the failing systemic RV and might be an important substrate for heart failure and arrhythmia. It is unclear if myofiber disorganization is due to hemodynamic factors, developmental problems, or both.

## Introduction

The myocardium is a highly anisotropic tissue, with myocytes (long axis approximately 10 times the transverse axis) organized into parallel bundles. The organization of myofibers appears to be important for heart function and electrical conduction. Early studies of myofiber organization were performed using histological sections [1, 2]. More recently diffusion-weighted (DW) cardiovascular magnetic resonance (CMR), a non-destructive technique that can yield a 3D data set, has been used to investigate myofiber architecture [3]. DW imaging uses the propensity of water molecules to diffuse within a tissue to characterize its structure. The principal eigenvector of the diffusion tensor measured in each voxel of the image indicates the direction of the long axis of myocytes in that voxel. The data from each voxel of the 3D data set are then combined to create a map of myofiber orientation (tractogram) over part or all of the myocardium. In addition to vector parameters, scalar parameters such as mean diffusivity (MD) and fractional anisotropy (FA), provide other information about myocardial structure [4–6]. MD measures the mean rate of water diffusion, with higher values indicating more unrestricted, extracellular space due, for example, to edema or cell lysis. FA describes the degree of anisotropy of the diffusion process and is thought to be a measure of tissue microstructural integrity. While this has been demonstrated in neural tissue, it is less clear in myocardium.

Pathological states, including some genetic cardiomyopathies as well as myocardial infarction, have been shown to be associated with abnormal tissue structure as indicated by abnormal MD and FA values as well as disarray of myofibers seen in tractograms [6, 7]. We hypothesized that myoarchitecture might be abnormal in some patients with congenital heart defects and heart failure and/or arrhythmia. In particular, we suspected that the architecture of a right ventricle (RV) serving as the systemic ventricle might be particularly affected, in part because of the bizarre gross appearance of these ventricles. We suspected that abnormal myoarchitecture might be an important contributor to the high prevalence of heart failure and arrhythmia seen in this subset of congenital heart disease patients late after surgical palliation [8, 9].

In this work, we tested our hypothesis that myofiber architecture is abnormal in the systemic RV of explanted, failing hearts using ex vivo DW CMR.

## Methods

### Patients

The explanted hearts from 3 patients with a systemic RV and from 1 patient with a structurally normal heart with acquired RV hypertrophy were examined. The families gave consent for the hearts to be used for research and the protocol was approved by the institutional review board (IRB) (IRBP00016280).

### Heart preparation

The explanted hearts were fixed (5% formalin and 50% ethanol) under 10 cm of water distending pressure and immersed in Galden® (Kurt J. Lesker Company, Jefferson Hills, Pennsylvania, USA) for imaging. Each heart was packaged in a sealed, close-fitting bag to minimize the distance between the CMR coil and the sample. Care was taken to remove microscopic air bubbles to reduce B1 field inhomogeneity so that susceptibility geometric and intensity distortion were minimized.

### Diffusion imaging

All imaging was performed on a 3 T CMR system (Skyra, Siemens Healthineers, Erlangen, German) with a pulsed gradient spin echo sequence and single shot echo planar imaging (EPI) readout pulse sequence. We have used the posterior part of the 64-channel head coil at the bottom of the sample and a 7 cm loop coil at the top of the sample to increase signal and coverage. Eddy current distortion was minimized by utilizing a twice-refocused spin echo sequence [10]. Scan parameters were adapted for each case to account for variations in heart size (Table 1). The main reason for different spatial resolution in each acquisition was due to the condition of the ex-vivo specimens. Depending on the post-mortem interval and fixation, each sample had a different CMR T2 contrast and total signal, leading to a different signal-to-noise (SNR) per unit time. CMR acquisition parameters were tuned to compensate for that and to allow robust DWI fitting for each sample.​ Higher spatial resolution images were also acquired with smaller specimens (Double-Inlet RV (DIRV), RV hypertrophy (RVH 'normal') to maintain details, while the number of averages was increased to compensate for loss of SNR associated with high-resolution imaging. The diffusion gradient schemes included a multi-shell (4 b = 0 images and 3 shells at b = 1000, 1500, 2000s/mm2 of 30 gradients each) and the CUSP90 Scheme [11], (12 b=0 images and 78 gradients with 400 s/mm2 < b < 3000 s/mm2) which allowed imaging with lower echo time (TE) than a multi-shell, providing a substantial SNR boost. EPI is highly sensitive to magnetic field inhomogeneities caused by susceptibility changes due to air bubbles in ex-vivo imaging. This results in severe distortion along the phase-encoding direction. First, susceptibility distortions were prospectively minimized by careful packaging of the specimens. Second, the number of phases encodes in the phase encoding direction was minimized by reducing field-of-view (FOV) in this direction for each case to reduce the amplitude of distortion caused by any residual microscopic bubbles. Finally, the entire gradient scheme was repeated with opposite phase encoding directions (anterior-posterior (AP) and posterior-anterior (PA), see Table 1) to allow estimation of the susceptibility-induced off-resonance field and correction of both geometric and intensity distortion using Topup [12]. A single average scan required ~ 40 min, with an entire specimen scan requiring 6–10 h depending on the size.

**Table 1:** Imaging Parameters

	Age	FOV (mm^2^)	Matrix size	Slices	Slice thickness	Resolution (mm^3^)	TE (ms)	TR (s)	Repetitions	Diffusion scheme
MA/AA	14 y	85 × 59	86 × 60	66	1.5 mm	1.5 × 1.5 × 1.5	101	13.3	9AP, 9PA	Multishell
DIRV	5 y	95 × 77	96 × 78	70	1.2 mm	1.2 × 1.2 × 1.2	91	13.0	8AP, 8PA	[6–11]
C-TGA	19 y	99 × 99	100 × 100	84	1.5 mm	1.5 × 1.5 × 1.5	89	15.9	6AP, 6PA	[6–11]
RVH ‘normal’	1 y	108 × 78	108 × 78	46	1.0 mm	1.0 × 1.0 × 1.0	96	8.6	20AP, 20PA	[6–11]

*Abbreviations*: *AP* anterior-posterior, *C-TGA* corrected TGA {S,L,L} heart, *DIRV* double-inlet right ventricle heart, *FOV* field of view; *MA/AA* mitral atresia/aortic atresia heart, *mm* millimeter, *ms* millisecond, *PA* posterior-anterior, *RVH* heart with normal anatomy but postnatal right ventricular hypertrophy, *s* second, *TE* echo time, *TR* repetition time

We characterized the tissue microstructure using diffusion compartment imaging (DCI) [13] estimated from the multi b-value acquisition. The most commonly used DW-CMR technique is diffusion tensor imaging (DTI) [14] which is well known to conflate the signal arising from the various tissue microstructures in each voxel by estimating a simplified single diffusion tensor, resulting in reduced specificity in describing the specific nature of pathological tissue changes. DCI, instead, considers that the signal in each voxel arises from a finite number of large-scale, microstructural environments in slow exchange, and uses a multi b-value acquisition to disentangle them. We considered a DCI model with one free diffusive component at each voxel to represent the contribution of free diffusion and one anisotropic component to characterize the orientation and diffusion characteristics of myofibers. Each compartment was modeled using a continuous, peak-shaped, statistical distribution of diffusion tensors [13], allowing us to capture both the average compartment diffusivity and its microstructural heterogeneity. The free diffusion was modeled mostly to account for partial voluming at the tissue interface. We chose in this work to focus our quantitative analysis on the source of anisotropy in the tissue, i.e. the myofibers.

### Image display and analysis

The orientation of each anisotropic compartment was displayed in glyph format and used for qualitative description of myofibers (MisterI, www.benoitscherrer.com/MisterI/) and for construction of tractograms (FiberNavigator) [15].

**Fig. 1:**
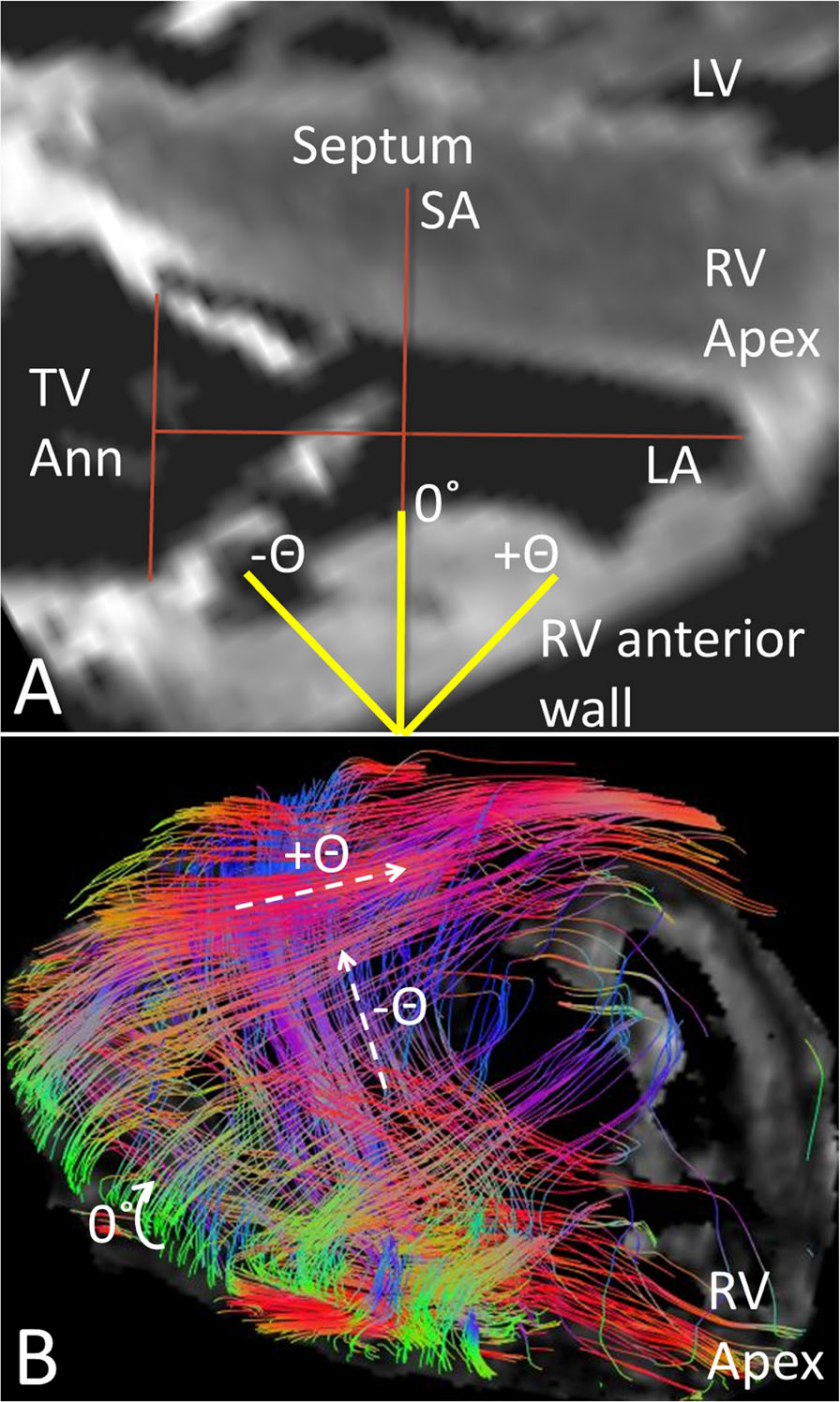
**a** Anterior CMR view of a normal right ventricle (RV) illustrating the convention we have used for helix angle as described in the Methods. First, the long axis (LA) of the ventricle was constructed as a line from the center of the tricuspid valve annulus (TV Ann) to the apex of the right ventricle (RV Apex). A short-axis plane (SA) was then constructed perpendicular to the long axis of the ventricle. The SA plane was considered 0°. Fibers running superiorly and toward the base were assigned a negative helix angle (−Θ) and those directed superiorly and toward the apex a positive helix angle (+Θ). LV – left ventricle; Septum – interventricular septum **b** Anterior oblique view of the MA/AA heart with a tractogram of the rightward-anterior wall of the RV shown. The red epicardial fibers (dashed arrow +Θ) are directed superiorly and toward the apex with a positive helix angle. In contrast, the blue endocardial fibers (dashed arrow –Θ) pass superiorly and toward the base with a negative helix angle. The green fibers at the acute margin (curved solid arrow 0°) are circumferential epicardial fibers. Color coding varies from sample to sample according to the scan direction and will be indicated in each figure showing glyphs and tractograms

The helix angle was computed across the ventricular wall in 5 evenly spaced locations using MisterI software. The long axis of the ventricle was constructed as a line from the center of the AV valve to the apex of the ventricle (Fig. 1). A short-axis plane was constructed perpendicular to the long axis and used as the 0° reference for the helix angle. The helix angle was measured as the angle between the principal eigenvector of the anisotropic compartment projected on a plane tangent to the epicardium and the short-axis plane of the ventricle. To our knowledge, there is no convention for describing the helix angle of the RV as there is for the left ventricle (LV) [7]. We assigned positive angles to fibers oriented superiorly and toward the apex when viewed from outside the ventricular wall with the heart in anatomic position, and a negative angle to fibers oriented superiorly and toward the base of the heart. We recognize that the myocardium is a syncytium composed of bundles of elongated myocytes joined by gap junctions and surrounded by perimysium rather than individual myofibers. We use the term myofibers to aid in the visualization and conceptualization of the diffusion streamlines displayed in the tractograms and tensor fields.

DCI allows characterization of diffusion scalar parameters such as FA and MD that are specific to each compartment, therefore referred to as compartment FA (cFA) and compartment MD (cMD). cFA and cMD values were computed at each voxel and average values were derived for each specimen as a whole and for each of their walls (free walls of the RV and LV for biventricular hearts and right and left walls for univentricular hearts).

### Histology

Blocks of ventricular myocardium were removed after imaging, sectioned, mounted and stained, photographed, and stored digitally for comparison with diffusion data. The heart specimen was imaged with computed tomography following removal of tissue samples and a 3D reconstruction created (MisterI). The diffusion images were then overlaid on the 3D image to localize the sites for comparison with histology.

### Statistics

The quantitative statistical analysis was carried out using Stata (StataCorp. 2015. Stata Statistical Software: Release 14. College Station, Texas, USA). We used 1-way ANOVA to test the significance of differences in global means among the 4 hearts. We then used a Mann-Whitney U test (non-parametric) for pairwise comparisons of the global means of the 3 systemic RV hearts with the RVH ‘normal’ heart and for pairwise comparisons of the 2 free walls of the univentricular hearts.

## Results

### Patients

**Fig. 2:**
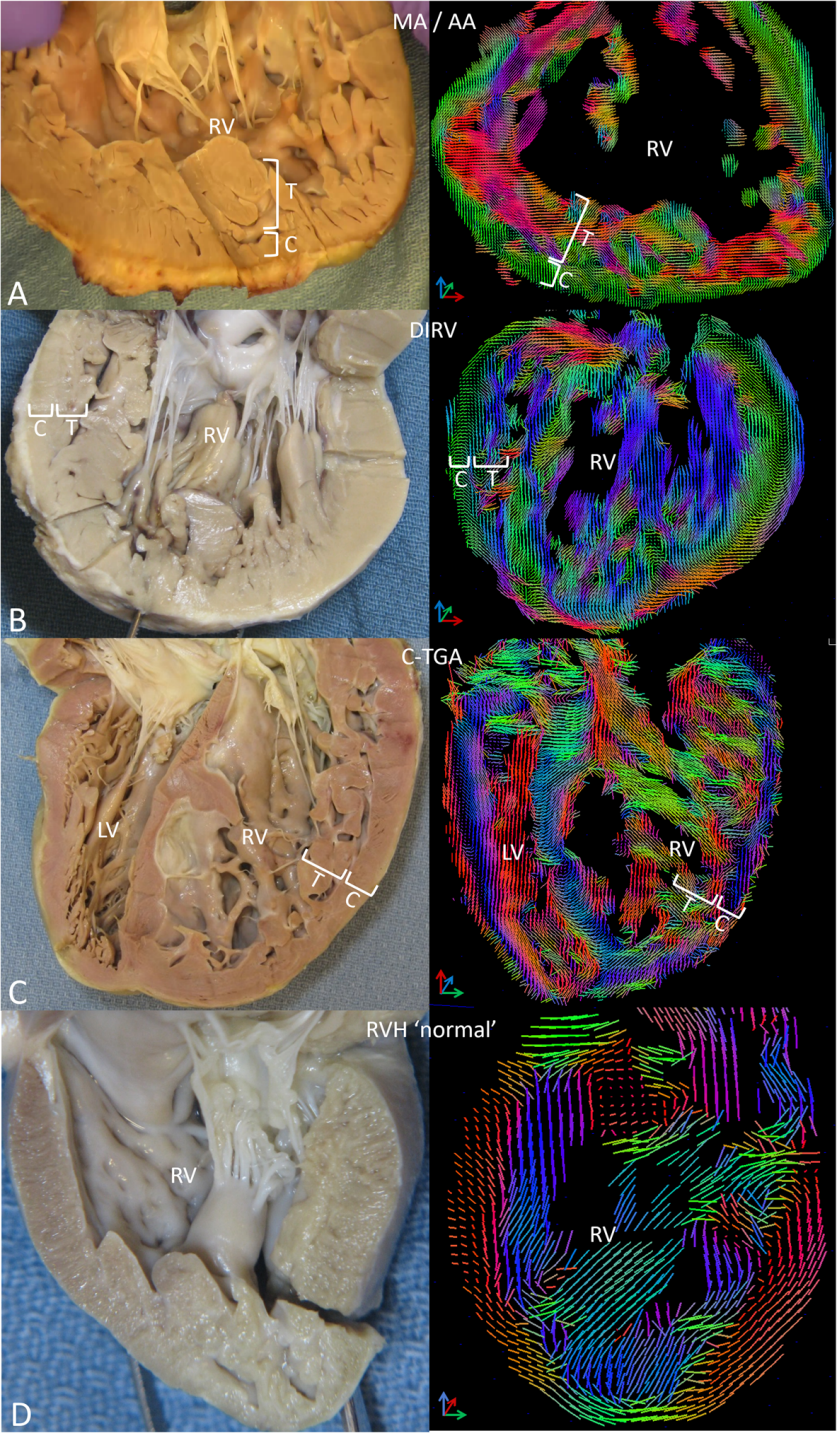
The formalin-fixed specimen (left) and DW CMR tensor field (right) in linear glyph format of explanted hearts from: **a**- a 16yo with mitral and aortic atresia (MA/AA), **b**- a 5 yo with double-inlet, double-outlet RV (DIRV), **c**- a 19yo with physiologically corrected transposition of the great arteries, atrio-ventricular and ventriculo-arterial discordance (C-TGA) and **d**- a 1yo boy with a structurally normal heart and postnatally acquired RV hypertrophy (RVH ‘normal’). In the 3 hearts with a systemic RV there is an outer compact, circumferential layer (C) and a thicker inner trabecular layer (T) with bundles of fibers running at large angles to each other. The RVH ‘normal’ heart was smaller than the other 3. Expanding the image to match the size of the other 3 hearts resulted in lower density of glyphs in the tensor field image. It has an external compact layer of nearly circumferential fibers with more longitudinally oriented, discrete fiber bundles internally, similarly to what has been reported for a normal RV but with thicker layers due to hypertrophy

**Patient 1** is a 14-year-old boy with mitral atresia/aortic atresia (MA/AA) who underwent Norwood palliation as a neonate, a bi-directional Glenn anastomosis at 10 months of age and fenestrated lateral tunnel Fontan operation at 3 years of age. The fenestration was closed (device closure) at 6 years of age. By 12 years he had developed increasing exercise intolerance, edema and ascites. Echocardiography showed moderate to marked global RV systolic dysfunction and mild-moderate tricuspid regurgitation (Supp Video 1, Fig. 2a). Exercise testing showed severely depressed peak workload and peak VO2. Cardiac catheterization showed low cardiac output (2.1 L/min/M^2^) and elevated RV filling pressure (18 mmHg) with pulmonary vein desaturation (88–92%). Symptoms progressed and he underwent orthotopic heart transplantation at age 14 years.

**Additional file 1**

**Patient 2** is a 5-year-old girl with DIRV and double-outlet RV (DORV) who underwent balloon septostomy and pulmonary valve dilation as a neonate because of cyanosis. At 6 months of age she underwent bi-directional Glenn anastomosis and atrial septectomy. At 2.5 years she underwent fenestrated lateral tunnel Fontan operation, with device closure of the fenestration 6 weeks later. By 5 years she had developed increased fatigue and abdominal pain with exercise. An echocardiogram showed severely depressed global RV systolic function with minimal left AV valve regurgitation (Supp Video 2, Fig. 2b). A catheterization showed normal cardiac output (3.1 L/min/M^2^), mean pressure in the Fontan circuit of 10–11 mmHg, wedge and RV filling pressure of 6 mmHg, and no coronary obstruction. She became increasingly symptomatic with limited activity, abdominal pain, and failure to gain weight. She underwent orthotopic heart transplantation at 5 years of age.

**Additional file 2**

**Patient 3** is a 19-year-old man with congenitally physiologically corrected transposition of the great arteries (C-TGA), atrio-ventricular and ventriculo-arterial discordance, who underwent closure of a ventricular septal defect at 3 months of age. By age 10 years he had begun to have reduced stamina. Echocardiography showed moderately decreased RV systolic function which progressed to severe global dysfunction by age 16 years (Supp Video 3, Fig. 2c). Exercise testing showed severely decreased peak workload and peak VO2. An implantable cardioverter-defibrillator was placed at age 16 years because of ventricular tachycardia. Cardiac catheterization showed a low cardiac output (1.9 L/min/M^2^), markedly elevated RV filling pressure (33 mmHg), pulmonary artery hypertension (1/2 systemic), and elevated systemic venous pressure (12 mmHg). Heart failure progressed leading to inpatient treatment with inotropic support and heart transplantation at age 19 years.

**Additional file 3**

#### RVH (‘normal’)

For comparison, a heart with postnatally acquired RVH was obtained at autopsy from a 1-year old boy who died from idiopathic pulmonary vein stenosis (Fig. 2d).

### Systemic RV

The systemic RV is markedly hypertrophied and often bizarrely shaped (Fig. 2a, b, c – left panel). An endocardial layer, comprising about 2/3 of the wall thickness, is composed of hypertrophied, intermeshing trabeculae and an epicardial compact layer of approximately circumferential myofibers is recognizable around most of the RV. Glyph representation of myofiber organization indicates that the outer compact layer is composed of parallel, approximately circumferential fibers while the trabecular layer is composed of bundles of fibers often running at large angles to each other (Fig. 2a, b, c – right panel, Supp Video 4, 5). In contrast, the structurally normal heart with RVH (RVH 'normal') has an external compact layer of nearly circumferential fibers with more longitudinally oriented, discrete fiber bundles internally (Fig. 2d, Supp Video 4, 5). This is similar to what has been reported for a normal RV [16], but with thicker layers due to hypertrophy.

**Additional file 4**

**Additional file 5**

**Fig. 3:**
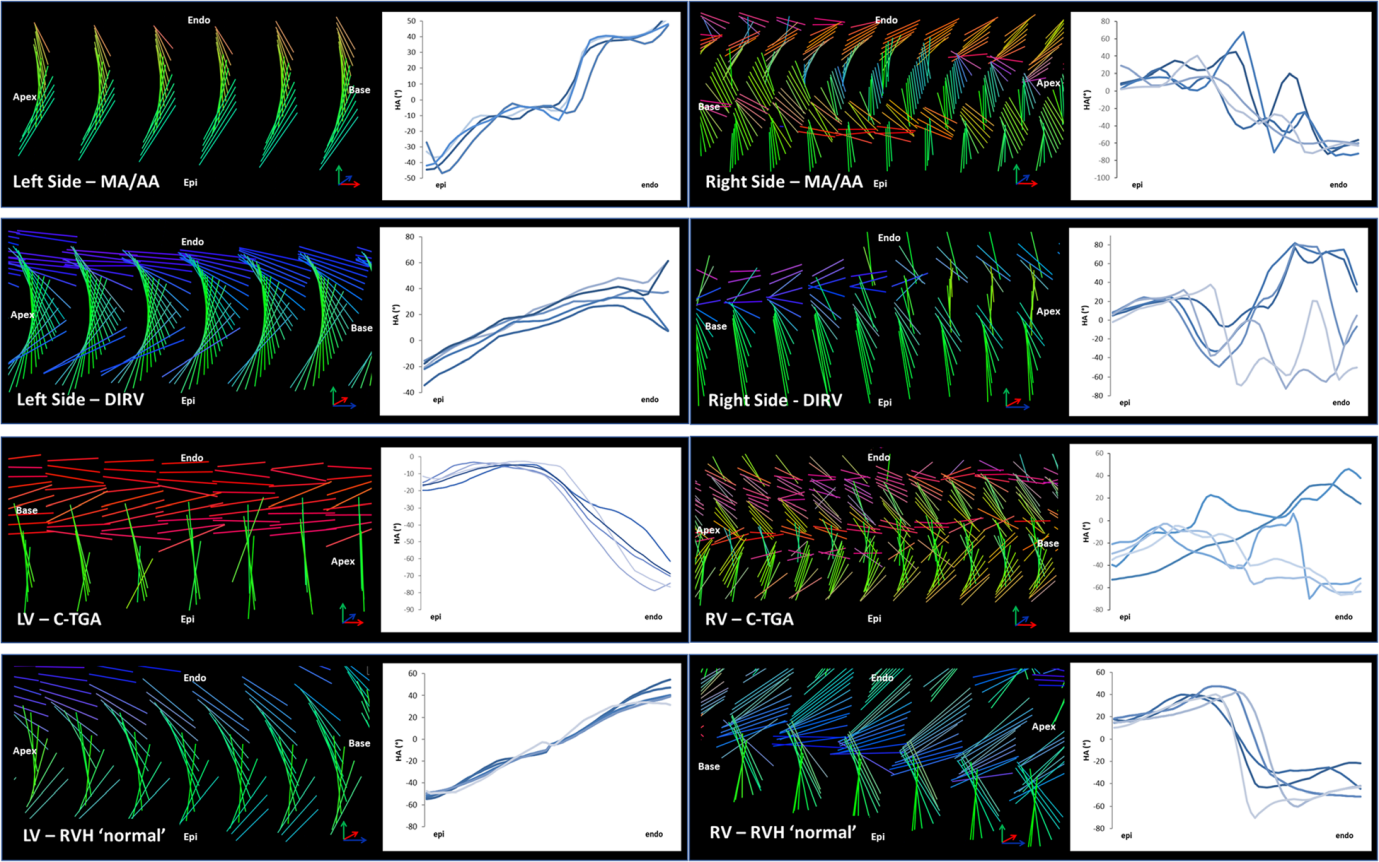
Graphs showing the transmural distributions of helix angle for the MA/AA heart (top), the DIRV heart (second), the C-TGA heart (third) and the hypertrophied but structurally normal RV (RVH ‘normal’) (bottom). Helix angle measurements were made at 5 sites distributed evenly around the free walls of the ventricles along a transmural line normal to a line tangent at the epicardial surface. Data were plotted from epicardial surface (left) to endocardial surface (right) with each line (varying shades of blue) representing one of the 5 positions around the ventricle

Plotting the helix angle of myofibers across the ventricular wall confirms the patterns described above (Fig. 3). In the structurally normal heart with RVH (RVH ‘normal’) (Fig. 3, bottom panel, right side), the helix angle of fibers in the outer half of the wall is between + 17.5° and + 47.3° (+ 27.4° ± 10.8°, mean ± SD), indicating fibers running nearly circumferentially but directed slightly superiorly and apically. There is an abrupt shift at midwall to a helix angle of − 70.5° (min) to + 40.4° (max) (− 35.5° ± 40.4°, mean ± SD), indicating endocardial fibers running more longitudinally, but directed somewhat superiorly and basally. This pattern is consistent with what has been described previously in the normal RV [16].

The patterns in the systemic RV are very different from the RVH ‘normal’ as well as from descriptions of the normal RV from the literature [16]. We also observed differences in the myoarchitecture between the 2 sides of the RV in the MA/AA and DIRV hearts which was most apparent on helix angle analysis. The wall on the left side of the MA/AA heart, presumably where the LV should have developed, is composed predominantly of compact myocardium (Fig. 3, top panel, left side; supplementary video 4). The helix angle progression across the wall is somewhat like the normal LV, − 18.7° ± 12.7° (mean ± SD)° at the epicardial surface and + 28.1° ± 20.7° (mean ± SD) at the endocardial surface, but the progression is not smooth as seen in the normal LV, with a plateau around 0° at mid wall. On the right side of the heart (presumptive RV free wall) (Fig. 3, top panel, right side), the outer compact layer has a helix angle of + 9.9° ± 11.6° (mean ± SD)°, indicating nearly circumferential fibers, slightly angled up and toward the apex, while the inner trabecular region showed marked dispersion of the helix angle, ranging from about − 74.6° (min) to + 67.8° (max) (+ 27.2° ± 32.0°, mean ± SD), consistent with myocardial fibers at large angles with each other. The fiber pattern in the DIRV leftward wall (Fig. 3, 2nd panel, left side) is also reminiscent of the normal LV, with progression of helix angles from − 34.2° to + 27.6° (+ 2.5 ± 14.1°, mean ± SD) at the epicardial surface to + 7.3° to + 61.5° (+ 32.5° ± + 10.7°, mean ± SD) at the endocardial surface, but without the plateau at midwall (supplementary video 5). In addition, there was some dispersion of helix angles near the endocardial surface. The pattern in the rightward wall (Fig. 3, 2nd panel, right side) was similar to the rightward wall in the MA/AA heart, but with even more dispersion of the helix angles in the inner 2/3 of the wall (Supp video 5), with progression from − 2.0° to + 27.3° in the epicardial layer (+ 16.2° ± 6.6°, mean ± SD), and from − 72.5° to + 81.9° in the inner, trabecular layer (+ 4.8° ± 43.8°, mean ± SD). The RV free wall in the C-TGA heart (Fig. 3, 3rd panel, right side) was similar but with more dispersion in the outer layer, although a suggestion of a crossing helical arrangement is noted, ranging from − 52.9° to − 1.8° (− 22.4° ± 14.1°; mean ± SD) in the epicardial layer and from − 70.0° to + 46.1°(− 19.5° ± 31.9°; mean ± SD) in the endocardial, trabecular layer (Supp video 6).

**Additional file 6**

### LV in C-TGA

Interestingly, the LV in C-TGA (Fig. 3, 3rd panel, left side) appears bilayered, with outer nearly circumferential fibers (ranging from − 19.9° to − 2.6°; − 8.8° ± 4.8°, mean ± SD) and an inner longitudinal layer more like the normal RV (ranging from − 78.8° to − 4.5°; − 41.0° ± 20.3°, mean ± SD), as opposed to a normal LV. The LV in the RVH ‘normal’ heart was structurally normal with normal wall thickness and showed the typical crossing helical pattern of the LV free wall (Fig. 3, bottom panel, left side). The helix angle progresses smoothly from about − 55.8° to − 2.9° (− 32.9° ± 14.0°, mean ± SD) at the epicardial surface to about − 11.3° to + 54.4° (+ 18,7° ± 15,7°, mean ± SD) at the endocardial surface.

### Small trabeculae vs large composite bundles

**Fig. 4:**
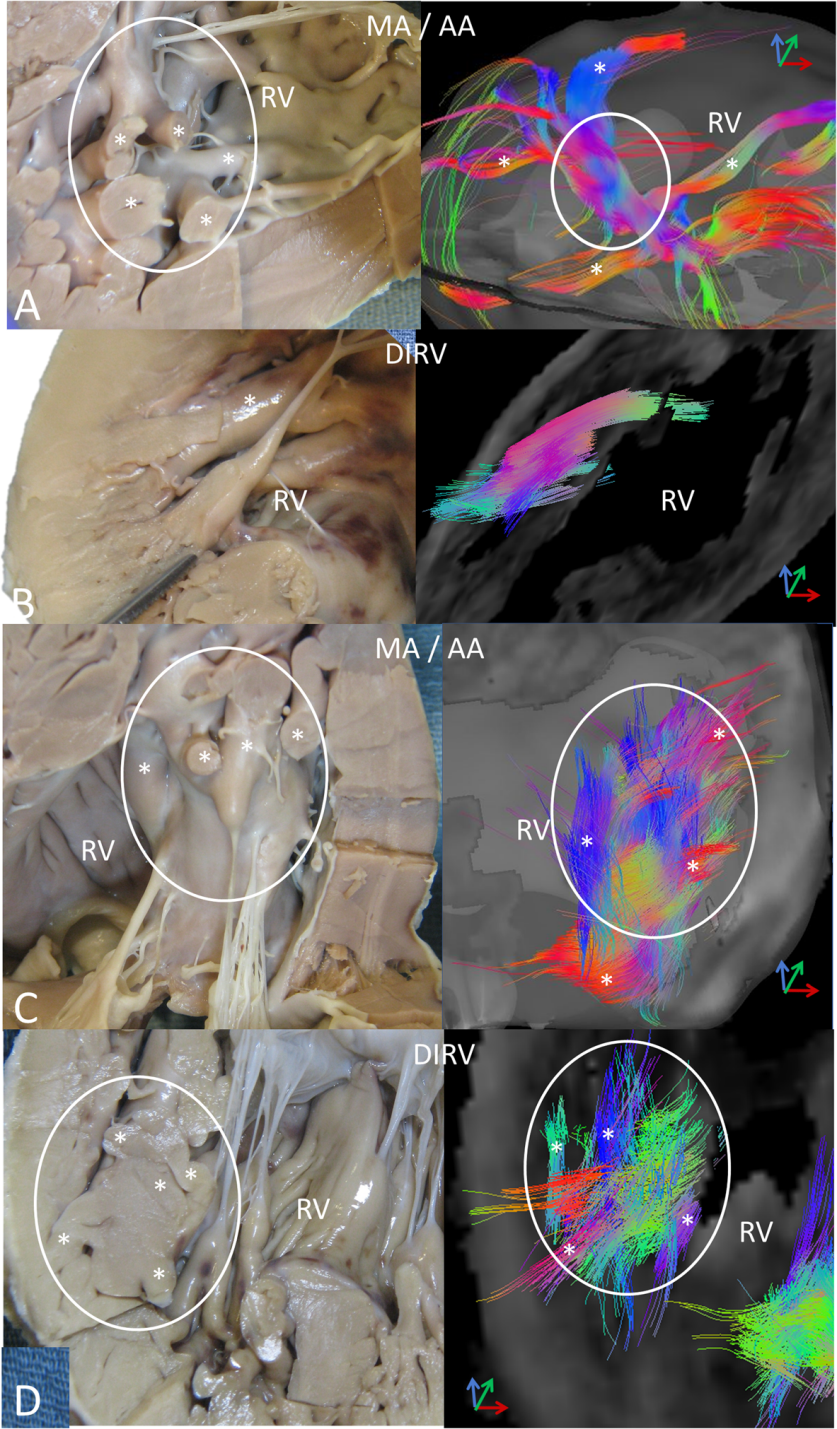
Myofibers in the systemic RV tend to be organized within individual, small trabeculae (*) and papillary muscles a few mm up to ~ a cm or so in diameter. In contrast, larger muscle bundles (enclosed in white ellipses), which are composites of the small trabeculae (*) and measure up to a few cms, appear disorganized with many crossing fibers. **a** – Parallel fiber tracts in small trabeculae (*) in the MA/AA heart. These small trabeculae cross each other as they converge into a larger bundle (ellipse). **b** – A small papillary muscle on the wall of the DIRV heart. **c** – A large muscle bundle (ellipse) in the MA/AA heart showing the many crossing trabeculae (*). **d** – A similar complex, large bundle (ellipse) in the DIRV heart

In the systemic RV, smaller muscle bundles are organized with parallel fibers (Fig. 4a, b). These are small papillary muscles or free wall trabeculations measuring a few mm to a cm or so. Some of these trabeculae and papillary muscles fuse together to form larger (up to a few cm), composite muscle bundles. In contrast to component trabeculations, fiber orientation in larger, composite bundles shows marked variability, largely between component trabeculae (Fig. 4c, d, Supp videos 7), which appears to account for the helix angle dispersion in the inner, trabecular portion of the wall.

**Additional file 7**

### Apical whorl

**Fig. 5:**
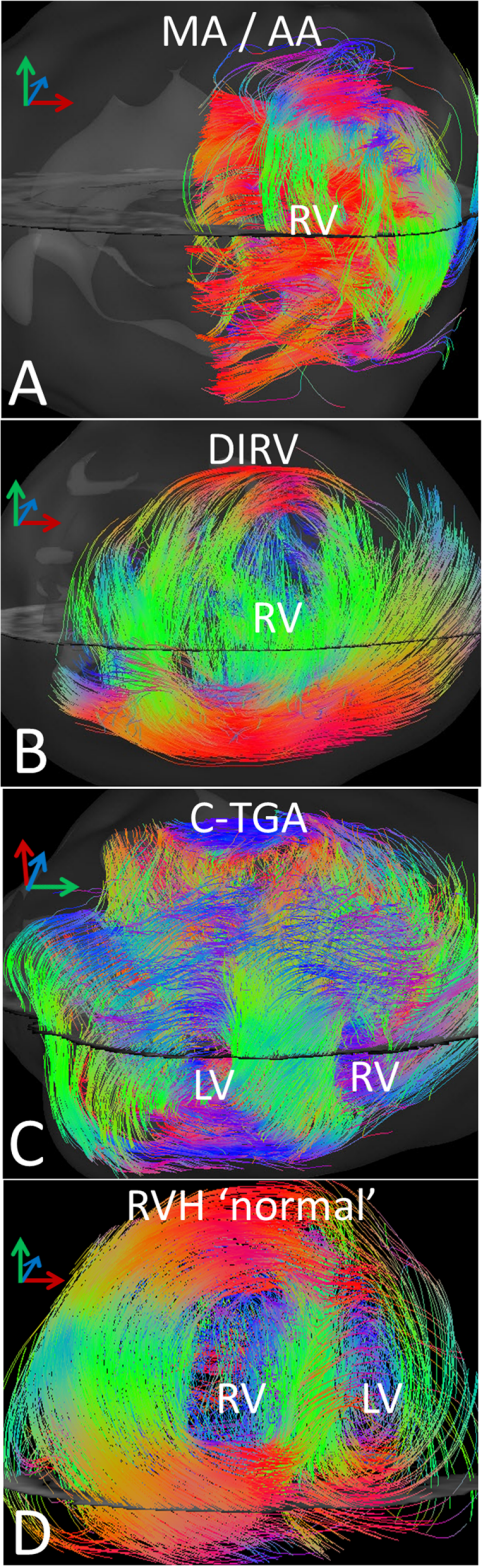
The apical whorl of the systemic RV is replaced by an incomplete whorl or even a chaotic net of fibers (Supp video 8). **a** –MA/AA heart showing absence of an organized apical whorl with disorganized, crossing fibers. **b** – DIRV heart has a more organized apex with a 2-part whorl and ingression of fibers to form longitudinal muscle bundles (blue). **c** – An inverted apex in C-TGA heart with a well-organized LV apical whorl (right-sided) and a smaller and incomplete RV whorl (left-sided). **d** – Apex of the hypertrophied structurally normal heart (RVH ‘normal’) showing a large, organized RV apical whorl and a smaller LV whorl where epicardial fibers take a curved course around the apex and ingress superiorly and inferiorly to form longitudinal endocardial fibers on the septum and free wall, respectively

The apical whorl of the RV is typically where circumferential epicardial fibers take a circular course around the apex and then ingress to form longitudinal endocardial fibers. Fibers ingressing superiorly tend to form longitudinal septal fibers while those ingressing inferiorly form free wall longitudinal fibers. The apical whorl of the systemic RV is abnormal with chaotic arrangement of fibers in some cases and an incomplete whorl in others (Fig. 5, Supp video 8). The apex of the MA/AA heart has no clear whorl, rather there is a meshwork of crossing fibers and no organized ingression to form longitudinal fibers. The apex of the DIRV heart has 2 partially formed whorls and some fibers ingress to form longitudinal fibers. The apex is left-sided in the C-TGA heart and the whorl is partially formed but disorganized with crossing fibers superiorly and inferiorly. In contrast, the apical whorl of the hypertrophied RV is well-organized and complete with normal ingression of epicardial fibers to form endocardial longitudinal fibers (Fig. 5, Supp video 8).

**Additional file 8**

### Whorls and vortices away from the apex

**Fig. 6:**
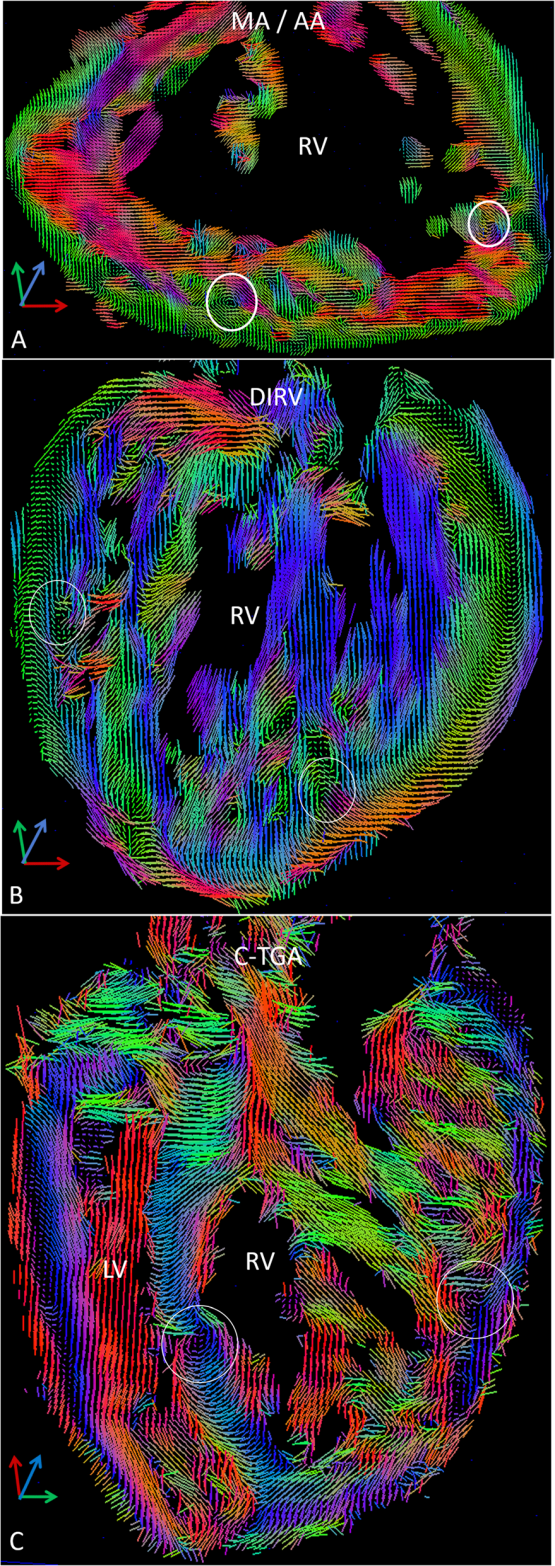
Vortexes of fiber bundles (white circles) are observed within the ventricular walls and trabeculations of the systemic RV, especially at the base of papillary muscles and large muscle bundles. Some are evident in planar tensor field images (**a** – MA/AA, **b** – DIRV, **c** – C-TGA). Others are more complex and are visible only in 3D tractograms (Supp video 9). We speculate that these could be a substrate for reentry arrhythmias

In addition, we observed myocardial whorls or vortices in multiple areas away from the ventricular apex in the systemic RV (Fig. 6,). Most appeared to be in a single plane and could be seen in the tensor field images. Others were more complex and could only be seen in 3D tractograms (Supp video 9).

**Additional file 9**

### Scalar parameters

Global cFA (Table 2) was significantly lower in structurally abnormal hearts (MA/AA, DIRV, C-TGA) compared to the structurally normal heart with RV hypertrophy (RVH ‘normal’). It was also lower in the right-sided wall of the 2 univentricular hearts (MA/AA, DIRV) compared to the contralateral wall. cMD (Table 3) was significantly higher in the DIRV and C-TGA hearts compared to the heart with acquired RV hypertrophy (RV ‘normal’) but significantly lower in the MA/AA heart. cMD was also lower in the right-sided wall of the DIRV heart but did not differ significantly between the 2 sides of the MA/AA heart. cMD was also lower in the right-sided wall of the DIRV heart but did not differ significantly between the 2 sides of the MA/AA heart.

**Table 2:** Global and local cFA

	Mean	SD	*p* value
			RVH ‘normal’ vs others
MA/AA	0.095	0.009	< 0.001
DIRV	0.188	0.038	< 0.001
C-TGA	0.180	0.018	< 0.001
RVH ‘normal’	0.324	0.096	N/A
MA/AA left side	0.098	0.005	< 0.001
MA/AA right side	0.091	0.014	
DIRV left side	0.173	0.003	< 0.001
DIRV right side	0.164	0.044

**Table 3:** Global and local cMD

	Mean	SD	*p* value
			RVH ‘normal’ vs others
MA/AA	0.000676	0.000013	< 0.001
DIRV	0.000719	0.000044	0.04
C-TGA	0.000984	0.000018	< 0.001
RVH ‘normal’	0.000700	0.000027	N/A
MA/AA left side	0.000688	0.000026	0.18
MA/AA right side	0.000688	0.000012	
DIRV left side	0.000813	0.000058	< 0.001
DIRV right side	0.000652	0.000040	

### Histology

**Fig. 7:**
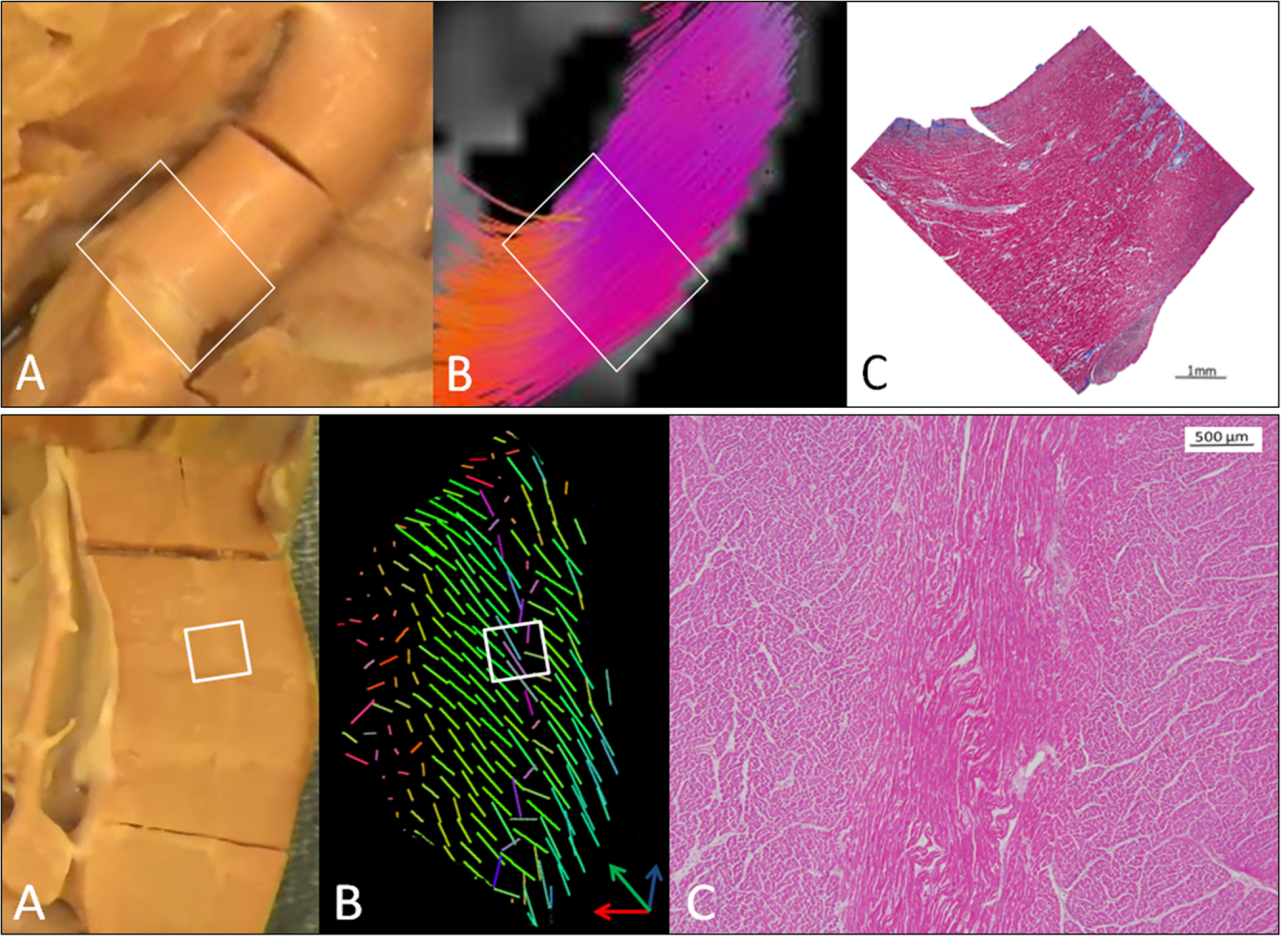
Upper panel – **a** - A muscle bundle from the MA/AA heart, **b** - the tensor field of the muscle bundle with linear glyphs showing parallel fibers, and **c** - an H&E stained section confirming the fiber organization. Lower panel – **a** – a section of the leftward wall of the RV in the MA/AA heart showing a thin, pale streak running through the white box. **b** – the tensor field in glyph format showing that the fibers on either side of the streak are circumferentially oriented (green) while the fibers in the streak are longitudinal (blue). **c** – An H&E stained section from the area of the white box confirming that the fibers to the left and right in the image are cut in cross-section (circumferential fibers) while those in the darker area corresponding to the streak are sectioned longitudinally

Fiber orientation by diffusion imaging closely matched histology (Fig. 7). Comparisons between histology and tensor field images or tractograms are qualitative because the streamline in the tractograms and the glyphs in the tensor field images represent 100 s or 1000s of myocytes in bundles. Consequently a 1:1 quantitative comparison is not possible.

## Discussion

We report the diffusion imaging of the myocardium in hearts with a systemic RV. As previously reported in hypertrophic cardiomyopathy [7], myocardial structural abnormalities are present in the systemic RV and likely result in inefficient contraction and ventricular dysfunction, as was present in our patients. These findings support our hypothesis that disordered myocardium contributes to heart failure in patients with a systemic RV.

Although the epicardial fibers were approximately circumferential as in the normal RV, there was much greater dispersion of helix angle in the inner trabecular layer. This layer comprises at least ½ of the ventricular wall. Myofiber disarray in such a large portion of the wall is very likely to result in dysfunction. cFA was significantly lower in the systemic RV compared with the RVH ‘normal’ RV indicating loss of structural integrity of the myocardium. cMD was significantly higher in 2 of these hearts compared to the RVH ‘normal’. This indicates increased extracellular space consistent with myocardial edema and/or fibrosis, both of which are associated with ventricular dysfunction and risk of arrythmias.

It is unclear if the RV apical whorl plays the same role as that of the LV, where it contributes to LV torsion, an important component of wall motion. The apical whorl was chaotic or incompletely formed in the systemic RV. If it is important for ventricular function, this could be another mechanism of dysfunction. Alternatively, the abnormal whorl could be simply a manifestation of myofiber disarray. Further, the myocardial whorls seen in the myocardium away from the apex could be the substrate of arrhythmia, another frequent complication of palliation of a systemic RV. Our findings support further studies of myocardial architecture in congenital heart defects.

In the longer term, in vivo diffusion imaging [17, 18] may be a groundbreaking tool for evaluating the suitability and for risk stratifying candidates for complex surgical treatment of congenital heart defects. The principal impediment to clinical use of diffusion imaging in the heart is motion created by the cardiac and respiratory cycles. The pulse gradient spin echo (PGSE) sequence used for ex vivo imaging is unsuitable for in vivo diffusion imaging because of its intrinsic susceptibility to motion-induced artifact. Consequently, other approaches have been employed. The most common sequence used to date for clinical imaging is the stimulated echo acquisition mode (STEAM). This approach can be implemented on standard imaging equipment without the need for high magnetic gradients. However, encoding occurs over two consecutive heart beats, during which the heart must be in the same location. This requires careful respiratory gating. Most reports have been in healthy, motivated subjects who were able to breath-hold or follow a visual feedback system to carefully control breathing. Both the requirements for two heart beats and for tight respiratory gating resulting in long imaging times make this approach not feasible for ill patients with little respiratory control and frequent arrhythmias. Another approach uses a spin echo sequence with high magnetic gradient strength to minimize the duration of diffusion encoding gradients relative to cardiac action thus limiting or abolishing motion-induced artifact. Unlike STEAM, this is carried out in a single heartbeat. Post-processing using principal component analysis to remove noise is followed by temporal maximum intensity projection to recover signal intensity lost to motion. Another spin-echo approach compensates for myocardial motion using myocardial strain analysis over the cardiac cycle (motion compensated spin-echo) [19]. Although this method appears to have advantages at high performance magnetic gradients, it does not appear to perform as well as STEAM at usual, clinical magnetic field gradients [19]. Despite significant progress to clinically useful cardiac DW CMR, substantial challenges remain, especially for ill patients with limited respiratory control and arrhythmia.

Our patients were 5, 14, and 19 years old at transplantation. Consequently, from our data it is impossible to determine if the abnormal myocardial architecture is an intrinsic feature of the systemic RV or if this is acquired from long-standing hemodynamic abnormalities. The organization of the RV wall with an inner trabecular layer and an outer circumferential layer is reminiscent of the organization of a normal RV [16, 20] suggesting that the myofiber disorganization is at least partly secondary to hypertrophy and chamber dilatation. However, an experimental study of induced RV hypertrophy (banding the pulmonary trunk of young swine to induce hypertrophy followed by DW CMR analysis of myoarchitecture) [16] and our case of postnatally acquired RV hypertrophy failed to demonstrate abnormal RV myoarchitecture. Perhaps the abnormal hemodynamics in utero, when myoarchitecture is developing, play an important role in this process. Mekkaoui et al. [21] examined the development of myofiber patterning in a series of human fetal hearts. Although they did not look specifically at the RV, the time course of patterning is likely to be similar to the LV. At 10 weeks of gestation, just after the completion of embryogenesis, myocytes are relatively isotropic so that a clear DW signal was difficult to obtain. The process of myofiber organization begins to develop between 14 and 19 weeks of gestation and continues even after birth. Examination of abnormal fetal and neonatal hearts will help to resolve these questions.

Our findings could have important implications for embryogenesis of these congenital defects. The differing patterns seen in the 2 sides of the systemic RV in the 2 hearts without a recognizable LV could be a clue to embryonic development, with varying sources of cells and developmental programs for the 2 sides of the heart even in these hearts with apparently only one ventricle.

Quantitative analysis showed reduced cFA in structurally abnormal hearts compared to a structurally normal heart with postnatal RV hypertrophy. These findings are consistent with what has been described for other cardiac diseases (ischemic or non-ischemic), in which loss of fiber orientation, fibrosis and disarray lead to a reduction of FA [4, 22]. Differences in cMD were less consistent, with cMD being higher in the DIRV and C-TGA hearts but lower in the MA/AA heart compared to the RVH ‘normal’ heart. Although further studies are needed to explain these phenomena, the differences in scalar parameters between structurally normal and abnormal hearts and between the 2 ventricles or the 2 sides of the univentricular hearts appears to result from microstructure alterations [22] and correspond to the abnormal organization described by fiber tracking. While cFA seems to be more correlated with the variance in fiber orientation, it is not clear whether cMD values could be influenced by the degree of edema which could be present in such hearts.

The only previous report of diffusion imaging in a systemic RV of which we are aware was in a living adult patient with transposition of the great arteries and an atrial switch operation [23]. Helix angle measurements were not specifically reported but the 3D reconstructions appear to show an external roughly circumferential layer and an inner layer with more dispersion, similar to our findings. Recently, the myocardial architecture in a single fetal heart at 20 weeks gestation with a complex heart defect including a systemic RV was evaluated using high-resolution x-ray phase contrast computed tomography [24]. In that heart the RV wall had a thin external circumferential layer and a thicker, internal trabecular layer with dispersion of the helix angle, similar to what we observed in older patients with failing hearts. This suggests that at least part of the myoarchitectural disarray that we have reported here is present in utero and is likely due to abnormal development.

### Limitations

First, our study is based on a very small number of cases. This reflects the limited availability of such hearts for study and is only an initial exploration of these abnormal hearts. Second, although the resolution of our DW CMR exams was higher than most reported studies in human hearts, each glyph or fiber tract represents hundreds or thousands of myofibers. Consequently, our data reflect the orientation of aggregate myocardial diffusion streamlines and not individual myofibers. Given the organization of the myocardium in these hearts into trabeculae, the flow streams that we describe appear to reflect the level of myocardial organization likely to be important for ventricular function. Third, we had no control over the phase of the cardiac cycle in which the heart arrested. We fixed the hearts mildly distended to standardize the conditions as much as possible. Finally, the heart with RV hypertrophy and no structural heart defect was from a significantly younger child than the other hearts. However, it represents the effects of postnatal hypertrophy on myofiber organization.

## Conclusions

Our work shows for the first time that the failing systemic RV in congenital heart disease is abnormally structured and, although it is not clear yet whether these alterations are genetically or hemodynamically determined, they could be the substrate for heart failure and arrhythmias affecting these patients.

## Data Availability

The datasets used and/or analyzed during the current study are available from the corresponding author on reasonable request.

## Abbreviations

AP: Anterior posterior
cFA: Compartment fractional anisotropy
cMD: Compartment mean diffusivity
CMR: Cardiovascular magnetic resonance
C-TGA: Congenitally physiologically corrected transposition of the great arteries
DCI: Diffusion compartment imaging
DIRV: Double-inlet right ventricle
DORV: Double-outlet right ventricle
DTI: Diffusion tensor imaging
DW: Diffusion weighted
EPI: Echo planar imaging
FA: Fractional anisotropy
LV: Left ventricle/left ventricular
MA/AA: Mitral atresia and aortic atresia
MD: Mean diffusivity
PA: Posterior-anterior
PGSE: Pulse gradient spin echo
RV: Right ventricle/right ventricular
RVH: Right ventricular hypertrophy
SNR: Signal-to-noise ratio
STEAM: Stimulated echo acquisition mode
TE: Echo time
